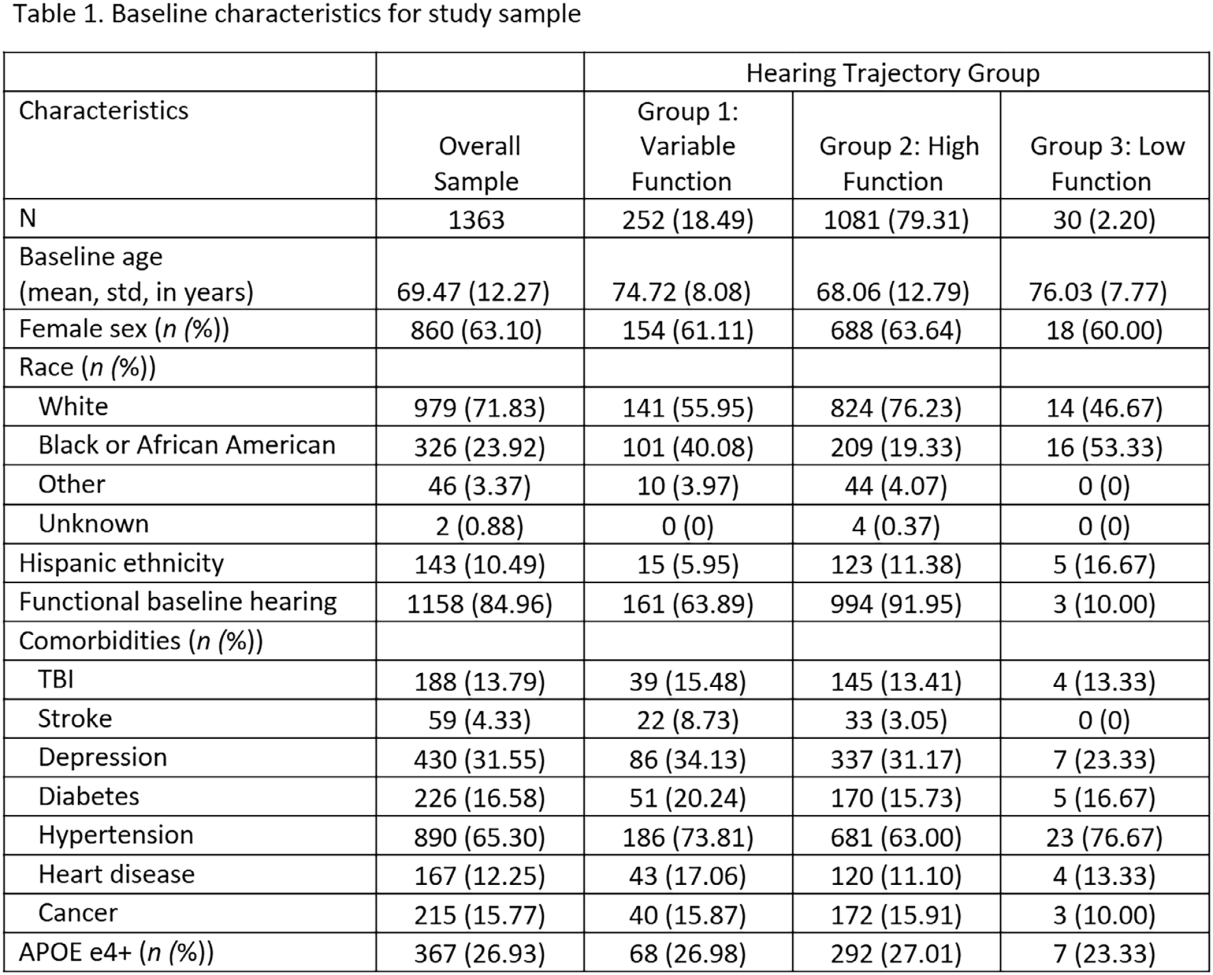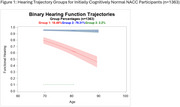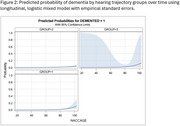# Later‐life hearing trajectories and dementia risk among initially cognitively normal adults: Results from the NACC Uniform Data Set

**DOI:** 10.1002/alz70860_096943

**Published:** 2025-12-23

**Authors:** Hannah R Speaks, Meredith S Duncan, Erin L. Abner

**Affiliations:** ^1^ College of Public Health, University of Kentucky, Lexington, KY, USA; ^2^ Department of Epidemiology and Environmental Health, University of Kentucky, Lexington, KY, USA

## Abstract

**Background:**

Hearing loss has an established association with dementia, but the longitudinal relationship has not been entirely understood. Characterization of hearing trajectories can inform strategies for prevention and treatment that may promote healthy brain aging. This study uses longitudinal data from the National Alzheimer's Coordinating Center (NACC) database to identify latent trajectories of hearing and their association with incident dementia and related risk factors.

**Method:**

We examined initially cognitive normal participants with at least three annual visits between 2005‐2023. Hearing status was dichotomized into functional and non‐functional hearing at each visit. Non‐functional hearing was defined by reported deafness or hearing disability, lack of ability to hear with hearing aids, or the inability to hear and complete neurological testing. Covariates with established associations with hearing or dementia were selected from the NACC Uniform Data Set (Table 1). We used group‐based trajectory modeling with age as the time scale to cluster individuals into latent trajectories of hearing status and assigned each participant to the trajectory group for which they had the highest posterior probability of membership. Summary statistics including chi‐square tests and ANOVAs were performed by trajectory group for each covariate and cross‐sectional dementia outcome. Then, we assessed the association of hearing trajectory group with longitudinal dementia status using a logistic mixed effects model with empirical standard errors.

**Result:**

The study sample (*n* = 1363) was 36.9% male with mean age of 69.5 years. During the study period, 80 participants developed dementia. Non‐functional baseline hearing status was associated with fewer years of education, greater prevalence of hypertension and stroke, and older baseline age. Three hearing trajectory groups were identified: a low function group (*n* = 30, 2.2%), a high function group (*n* = 1081, 79.3%), and a variable hearing loss group (*n* = 252, 18.5%). Hearing group membership was associated with ethnicity and heart disease in addition to age, education, hypertension, and stroke. Hearing trajectory group was not associated with longitudinal dementia outcomes (F (2, ∞) =0.82, *p* = 0.43).

**Conclusion:**

This study suggests that hearing trajectory over time may provide different insights compared to baseline hearing status, potentially serving as a proxy for confounding factors influencing both hearing loss and dementia.